# P31 An observational prospective audit on the use of vancomycin and ciprofloxacin in the treatment of severe community-acquired pneumonia in penicillin-allergic patients in NHS Lothian

**DOI:** 10.1093/jacamr/dlae136.035

**Published:** 2024-08-23

**Authors:** Zsofia Blair, Esperanza Palenzuela, Simon Dewar

**Affiliations:** Regional Infectious Diseases Unit, Western General Hospital, NHS Lothian, UK; Regional Infectious Diseases Unit, Western General Hospital, NHS Lothian, UK; NHS Lothian Antimicrobial Management Team, Western General Hospital, Edinburgh, UK; Regional Infectious Diseases Unit, Western General Hospital, NHS Lothian, UK; NHS Lothian Antimicrobial Management Team, Western General Hospital, Edinburgh, UK

## Abstract

**Objectives:**

To assess the current standard of care in NHS Lothian compared with guidelines for patients prescribed vancomycin and ciprofloxacin in severe community-acquired pneumonia (CAP) with penicillin allergies.

**Methods:**

In total, 182 patients were analysed over 8 weeks. Data were collected from online prescribing records and clinical notes. A total of 18 patients met the criteria of correct antibiotic combination and clinical indication. Data were collated and analysed using Microsoft Excel.

**Results:**

*Vancomycin*: Levels were taken in 11 out of 18 patients, of which 8 reached therapeutic levels in a median duration of 2 days. First therapeutic levels: median 8.5 mg/L, range <4–15.8 mg/L. *Ciprofloxacin*: 83% of patients received ciprofloxacin orally; 72% of patients were aged >65 years and 39% were co-prescribed oral steroids. No patients had documented fluoroquinolone counselling. *Antibiotics*: 33% of patients were stepped down to oral doxycycline. The total mean duration of antibiotic therapy was 7.3 days. *Allergies*: One documented as ‘anaphylaxis’. Eight unsure of reaction/unspecified in medical notes. Three received penicillin recently with no adverse effect. One penicillin delabelled during admission.

**Conclusions:**

Patients on average received longer than recommended total antimicrobial treatment. Only one third received the recommended IVOS option, however this may have been influenced by other factors such as positive microbiology. In the majority of patients, in which vancomycin levels were taken/required, therapeutic levels were achieved in 2 days. Most patients received ciprofloxacin orally, suggesting that patients were well enough to not necessitate IV route. This opens the possibility of recommending other oral antibiotics with high oral bioavailability as alternatives (e.g. levofloxacin, doxycycline). Additionally, patients were not counselled on the potential adverse effects of ciprofloxacin as recommended by MHRA guidance, despite almost three quarters of patients having factors (i.e. elderly, on steroids) placing them at greater risk of adverse effects. However, the benefits of giving treatment in severe CAP may outweigh these risks. This audit highlights that practice varies from the NHS Lothian guidelines. It raises the question of whether alternative antibiotic therapies should be considered for first line in penicillin allergic patients, such as levofloxacin used in the rest of Scotland. However, given the new MHRA guidance on limitation of fluoroquinolones, it may be that other health boards also consider changes to their guidelines. Education regarding fluoroquinolone use should be provided for clinicians and patients to ensure that the risks are included in decision-making. Focus should be put on reducing the number of patients with inappropriate penicillin allergy labels.

